# Genetic Diversity Analysis of Banana Cultivars (*Musa* sp.) in Saudi Arabia Based on AFLP Marker

**DOI:** 10.3390/cimb45030116

**Published:** 2023-02-22

**Authors:** Fatmah Ahmed Safhi, Salha Mesfer Alshamrani, Dalal Sulaiman Alshaya, Mohammed A. A. Hussein, Diaa Abd El-Moneim

**Affiliations:** 1 Department of Biology, College of Science, Princess Nourah bint Abdulrahman University, P.O. Box 84428, Riyadh 11671, Saudi Arabia; 2 Department of Biology, College of Science, University of Jeddah, Jeddah 21959, Saudi Arabia; 3 Department of Botany (Genetics), Faculty of Agriculture, Suez Canal University, Ismailia 41522, Egypt; 4 Department of Plant Production (Genetic Branch), Faculty of Environmental Agricultural Sciences, Arish University, El-Arish 45511, Egypt

**Keywords:** banana cultivars, Fluorescent-AFLP, germplasm, DNA fingerprinting, Saudi Arabia

## Abstract

Banana plantation has been introduced recently to a temperate zone in the southeastern parts of Saudi Arabia (Fifa, Dhamadh, and Beesh, located in Jazan province). The introduced banana cultivars were of a clear origin without a recorded genetic background. In the current study, the genetic variability and structure of five common banana cultivars (i.e., Red, America, Indian, French, and Baladi) were analyzed using the fluorescently labeled AFLP technique. Nine different primer pairs combinations yielded 1468 loci with 88.96% polymorphism. Among all locations, high expected heterozygosity under the Hardy–Weinberg assumption was found (0.249 ± 0.003), where Dhamadh was the highest, followed by Fifa and Beesh, respectively. Based on the PCoA and Structure analysis, the samples were not clustered by location but in pairs in accordance with the cultivar’s names. However, the Red banana cultivar was found to be a hybrid between the American and Indian cultivars. Based on Φ_ST_, 162 molecular markers (i.e., loci under selection) were detected among cultivars. Identifying those loci using NGS techniques can reveal the genetic bases and molecular mechanisms involved in the domestication and selection indicators among banana cultivars.

## 1. Introduction

Among the edible, vegetatively propagated, monocotyledonous, and herbaceous species of *Musa*, banana and plantain (*Musa* sp.) belong to the Eumusa section of the genus Musa, family Musaceae and order Zingiberales [1]. Bananas and plantains rank fourth after cereals in importance as food sources in many developing nations [2]. One hundred two million hectares of banana farms are found in humid tropical and subtropics in the Americas, Africa, Asia, and Europe, extending to Australia and Europe [2]. Numerous countries in Asia, Africa, Latin America, and the Pacific Islands rely on banana production for a large portion of their economies. There are about 145 million tons of banana production, of which only a few million tons are exported. The banana is, without a doubt, a staple food for millions of tropical residents [2,3]. There are many nutrients and carbohydrates in bananas and plantains, including carbohydrates, minerals, and vitamins [4,5]. Unlike other fruit crops, it grows faster than other perennials and produces fruit throughout the year. In banana cultivation, micropropagation or suckers are used for asexual propagation [6].

Unlike their wild relatives, cultivated bananas grow without pollination. Fantastic collections of parthenocarpic mutants have primarily been made by farmers and multiplied and distributed by vegetative propagation of spontaneously occurring mutants [7]. During the initial domestication process, a relatively limited portion of the genetic diversity of wild banana species was used [8]. It is essential to know about the genetic diversity and agroecological adaptations of *Musa* to address contemporary food security needs. Clone identification and taxonomic studies have relied heavily on morphological and agronomic characteristics [9,10].

Two wild species in the section Eumusa produce different genotypes: *Musa acuminata* (AA) and *Musa balbisiana* (BB). They are classified into other genomic groups, including AA, AB, and BBs classified as diploids, AAA, AAB, ABB, and BBBs classified as tetraploids, resulting from interspecific hybridization between *M. acuminata* and *M. balbisiana* [11]. Several unifying characteristics were observed in morphological studies of *Musa* species. Hybrid cultivars and wild types exhibit complex genome structures and phylogenetic relationships that require further investigation. Banana cultivation is susceptible to pests and diseases because of its narrow genetic base [12]. Further, abiotic stresses caused by global warming and climate change exacerbate this situation [13]. In order to boost banana productivity, identifying genotypes with high potential is crucial [14].

It is common practice in plants to use molecular markers to identify genetic differences in germplasm, identify duplicate accessions, and test for genetic fidelity [3]. The availability of molecular markers, particularly polymerase chain reactions (PCR)-based techniques, has led to the evaluation of *Musa* species’ genetic diversity. For example, the application of random amplified polymorphic DNA (RAPD) techniques, which provide helpful information and new insights into the taxonomy [15], restriction fragment length polymorphism (RFLP) [16], sequence-related amplified polymorphism (SRAP) [17], and microsatellites or simple sequence repeats (SSRs), inter-simple sequence repeats (ISSRs) [18]. The AFLP method combines the convenience of polymerase chain reaction (PCR)-based fingerprinting with the reliability of restriction-based fingerprinting [19,20]. Furthermore, AFLP allows high-resolution genotyping by rapidly generating hundreds of highly reproducible DNA markers [21]. This study investigated the genetic d and genetic relationships of banana cultivars with unknown genomic groups, introduced into three locations in Jazan, southeast Saudi Arabia.

## 2. Materials and Methods

### 2.1. Sampling Site

The study was performed in three districts of one department of the southwestern region of Jazan province in Saudi Arabia (the Fifa mountains, Dhamdh governorate, and Beesh town). Banana cultivars were collected from farms in the main banana-growing agroecological zones of the country. The agroecological zone of the southwestern regions of Saudi Arabia is characterized by three agroclimatic zones and ten subzones defined by geographic location and topography that differ in rainfall and air temperature [22]. High altitudes are characterized by lower temperatures and higher rainfall (400–450 mm per year), making vegetation more diverse [23].

### 2.2. Sample Collection

A total of eight *Musa* species and subspecies were used in this study. Three samples of fresh banana leaves of each cultivar were collected from the field, packed in plastic bags, labeled with a site code, and kept in iceboxes until examination. To avoid sampling duplication from the same individual, we did not sample plants located directly next to each other (Table 1).

### 2.3. DNA Extraction

According to the manufacturer’s instructions, plant genomic DNA was extracted from leaf samples using the WizPrep™ gDNA Mini Kit (Wizbiosolutions Inc, Seongnam, Republic of Korea) with a final elution volume of 50 mL. To check the DNA quality, we visually tested 5 uL of each sample by 1% gel electrophoresis. DNA appears as sharp bands when visualized under UV light using the Ingenius3 Gel documentation system (Syngene, UK). Extracted DNA was stored at −20 °C until required for PCR.

### 2.4. AFLP Protocol

AFLP analysis was carried out following the method of Vos et al. [24], with one modification in the labeling type, as primers were labeled fluorescently rather than radioactively labeled. All primers and adaptors were synthesized by Eurofins, Hamburg, Germany (Table 2). Samples were successfully tested with six different selective PCR combinations. The original PCR protocol was followed without modification. Visualization of the amplified products was performed by a private service using an ABI3730 DNA analyzer (Applied Biosystems, Waltham, MA, USA) with a size standard GS500-LIZ (Macrogen Fragment Analysis Service, Republic of Korea).

### 2.5. Data Analysis

Peak Scanner^TM^ (Applied Biosystems, USA) and Raw Geno V2 (Applied Biosystems, USA) were used to automate the AFLP scoring. The band-binary criterion was applied to the analysis of the AFLP data as the detected bands were codified as 1 when present and 0 when absent. As the total number of samples equals 8, thus a single sample frequency = 12.5%. Bands with a frequency of >87% or <13% are often uninformative or misleading when included in the analyses [25,26] and were, therefore, excluded from further analysis using FAMD 1.31 software [27].

The Bayesian clustering method was applied by using Structure V2.2 [28] to investigate the genetic structure. Triple independent simulations were performed per each assumed number of sub-populations K (tested K = 1 to 5). Parameters were set as the following burn-in period of 10,000 out of 100,000 MCMC iterations, and the admixture ancestry model was set on. Analysis of molecular variance (AMOVA) was performed to test the population genetic differentiation using Arlequin V3.5 [29]. The significance of Φ_ST_ was tested with 10,000 permutations for the detected AFLP loci.

## 3. Results

### 3.1. Fragment Analysis and Band Scoring

**Figure 1:**
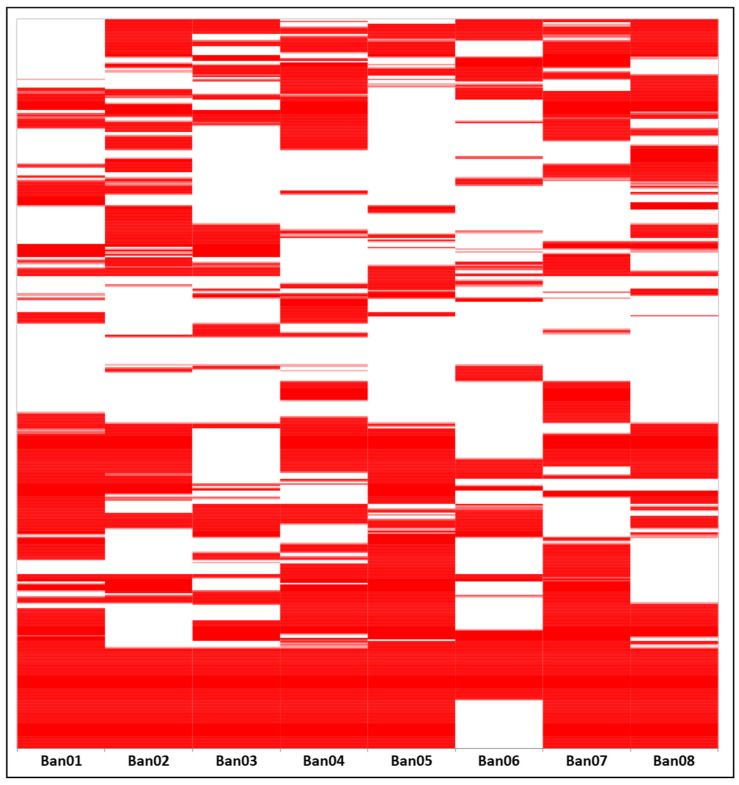
Heatmap of the binary scored bands for nine primer pairs applied to eight banana samples.

PCR amplification and fragment detection were successful for nine AFLP selective primer pairs. Among primer pairs, the average scored bands were 163 ± 35 bands ranging between 50 and 674 bp with an average size of 250 ± 78 bp (Supplementary Table S1). A weak significant negative correlation was found between fragment sizes and frequencies (r = −0.20; *p* < 0.00). Band scoring yielded a total number of 1468 bands with 162 monomorphic ones (88.96% polymorphism) for all primer pairs applied to the eight samples (Figure 1). After filtration, 136 loci (a band uniquely found in one sample, frequency below 13%) were removed to avoid bias, and 162 loci (locus found in all samples except for one, frequency above 87%) were removed and considered monomorphic. A total of 1008 loci were retained for further analysis.

### 3.2. Genetic Polymorphism and Diversity

Polymorphic bands for each location were 963, 862, and 571 for Dhamadh, Fifa, and Beesh areas, respectively. The effective number of alleles (n_e_) for all bulked samples combined was 1.46 ± 0.006. The expected heterozygosity under Hardy–Weinberg assumption (H_e_) for all bulked samples combined was 0.249 ± 0.003. Samples from Dhamadh scored the highest n_e_ (1.525 ± 0.01) and the highest H_e_ (0.292 ± 0.006) when F_IS_ = 1. Samples from Beesh yielded the lowest n_e_ (1.39 ± 0.013) and the lowest H_e_ (0.195 ± 0.006), while samples from Fifa scored 1.47 ± 0.010 for n_e_ and 0.261 ± 0.006 for H_e_ (Table 3).

### 3.3. Population Structure

**Figure 2:**
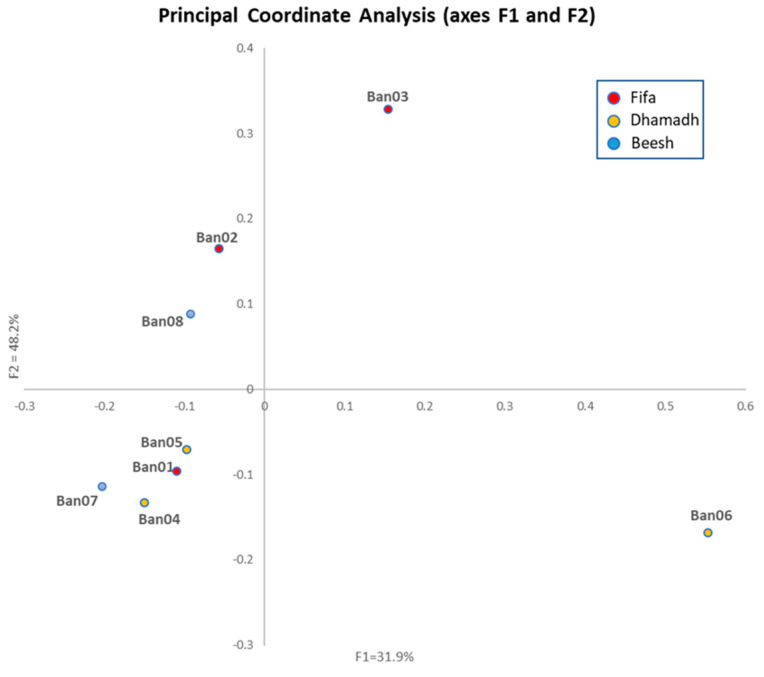
PCoA based on the Jaccard genetic distance matrix among the eight banana samples.

The dissimilarity genetic distance was calculated using the Jaccard coefficient; the distance ranged from 0.483 to 0.812. The two samples, Ban03 and Ban06, showed the highest dissimilarity values and were considered the most distant among all (Table 4). The principal coordinate analysis (PCoA) based on Jaccard genetic dissimilarity matrix showed non-location orientation. The demonstrated variation was between 31.9% (axis F1) and 48.2% (axis F2). The analyzed samples were clustered in pairs: Ban01 and Ban05, Ban04 and Ban07, both were clustered in the negative (x, y) quartile, the Ban02, and Ban08 in the negative x, positive y quartile, except for Ban03 plotted in the positive (x, y) quartile at a distance from Ban06 in the positive x, negative y quartile (Figure 2).

**Figure 3:**
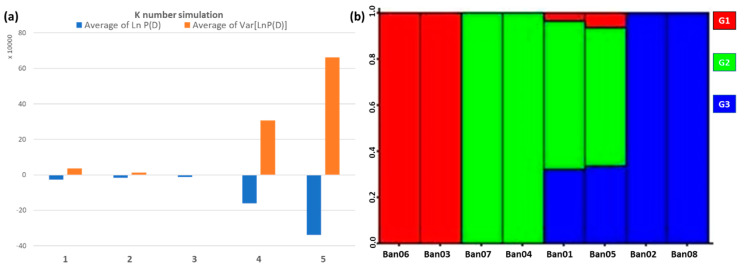
Bayesian inference of AFLP binary data using Structure software. The triplicates average for each K number was used to estimate Ln P(D) average and variance (**a**). The bar plot of the diversity portion for each of the eight samples is based on the three detected groups (k = 3; (**b**)).

The average estimated Ln probability score with the lowest variance was calculated for sub-population number K = 3, indicating that the observed samples most probably originated from three sub-groups (Figure 3a). Again, the sample structure was not clustered by location. Group 1 defines Ban03 and Ban06 samples with 100% homogenized diversity both are two different cultivars, the Baladi and French cultivars, respectively. Group 2 represents Ban04 and Ban07 samples with 100% homogenized diversity; both samples are of the same cultivar (American cultivar). Finally, group 3 defines Ban02 and Ban08 samples with 100% homogenized diversity; both samples are of the same cultivar (Indian cultivar). The only two samples that showed heterogeneous diversity were Ban01 and Ban05 samples, both are known as the Red banana cultivar; both samples showed the highest diversity portion of group 2, followed by group 3 and a minimal portion from group 1, reflecting a hybrid status mainly occurred between the American and Indian cultivars (Figure 3b).

### 3.4. Genetic Differentiation and Geographical Influence

The genetic differentiation was tested using AMOVA to measure the changes in the pairwise differentiation of the Φ_ST_ among the studied location and the cultivars. A very low Φ_ST_ of 0.07 among locations was detected, partitioned into a 93% genetic variation originating within locations, while 7% of the genetic variation occurred among locations. On the other hand, a much higher Φ_ST_ of 0.28 among cultivars was detected, partitioned into a 71.05% genetic variation originating within groups, while 28.95% of the genetic variation occurred among cultivars (Table 5). Based on the F_ST_ for each locus compared to the observed heterozygosity, 162 outlier loci were detected, differentiating all cultivars and considered loci under selection among cultivars (Supplementary Table S2). The AMOVA test then scored the maximum ΦST value of 1.00, as of 100% genetic differentiation originating from the differences between the cultivars and none within each (Table 5).

## 4. Discussion

Future research directions may also be highlighted. Recently, banana cultivations were established in Jazan province, a temperate region in the southeastern parts of Saudi Arabia. In several surveys related to banana cultivation in the Middle East, Saudi Arabia was never considered (e.g., de Langhe [8]). However, nowadays, initiatives to increase banana cultivation have been reported (e.g., a 100,000 banana-trees cultivation project was started by local businesswomen in Jazan [30]). The huge number of imported cultivars has drawn the scientific community’s attention to study and analyze them, especially at the genetic level. Using DNA fingerprinting techniques combined with botanical and physiological assessments would provide a clear base for selection procedures and biological maintenance. Application of DNA fingerprinting on banana plants were previously reported, whether to identify genotypes among wild species and cultivars [31,32], to estimate genetic diversity among cultivars [33] or genotypes [34], to resolve the link between genotypes and morpho-based classification [21], or to identify of duplicate accessions and genetic fidelity testing [3].

A high number of variable markers is possible with the AFLP technique, allowing genome-wide analysis of genetic variability. In our study, based on nine AFLP primer pairs combinations, 1468 loci were detected, compared to Opara et al. [35], who yielded 1094 loci when applied 12 AFLP primer pairs combinations to study local banana cultivars in the southern region of Oman. A comparison confirms the reproducibility of the used combination in our analysis, as a lower number of combinations yielded a higher number of loci. In an additional study, 22 AFLP primer pairs applied on 21 accessions yielded 485 bands only with 46.18% polymorphism (e.g., Ahmad et al. [36]). Thus, choosing the primer pairs combinations is critical to saving time and cost while improving the marker reproducibility and robustness. Based on the high reading output and extensive statistical analysis, the genetic variability of the samples was expected to be more clearly reflected. The likelihood of detecting markers under selection is relatively high, either directly or because they are located near genes under selection. The mean expected heterozygosity under Hardy–Weinberg assumption (He) was 0.249, regardless of the unequal diversity levels detected among the locations, which reflect a high diversity level among the samples. In a similar study, Wang et al. [37] detected high levels of genetic diversity for the wild banana progenitor *M. balbisiana* population, where a similar He of 0.241 was estimated, even though wild specimens usually record much higher diversity than the cultivated ones [36].

Molecular data consisting of unlinked markers are used by Structure software to infer population structure using model-based clustering. In Jazan locations, a genetic structure was detected, even though it was proven to be influenced by the genetic background of the cultivars rather than the sampling locations. Patterns of phylogeography have been tested for banana plants in China by Ge et al. [38], and all the genetic diversity analyses confirmed the significant geographical structuring when comparing wild to cultivated banana populations. The samples of the Red banana cultivars showed mixed portions of other groups (inferred by color). It is normal to observe traces of other cultivars’ genetic diversity, possibly due to the banana’s ancestral origin. The heterogeneity is based on the American and Indian cultivars with almost an equal portion, suggesting a clear hybridization event between both cultivars. On the other side, genetically related samples in group 1 were from different geographical locations and cultivars, known as the Baladi and the French cultivars. While they originate from distant locations, both cultivars showed the same similarity membership coefficient (i.e., a value that assigns a sample to a particular group). However, the PCoA clarified the genetic distance among both as unequal cultivars, proving the importance of complementing the structure analysis with PCoA analysis to resolve the correct genetic clustering [35,36].

There is increasing interest in identifying genes or outlier loci that underlie adaptations to different factors in several species or in finding signatures of selection and domestication [39,40,41]. Outlier loci are revealed when populations differ at specific markers [40,42]. In the current study, 162 outliers were detected, and those loci participated in the development and selection of banana cultivars, which were indeed found to exhibit increased differentiation among locations along with no genetic variability detected within cultivars. Similar studies confirmed the potential of the AFLP technique to detect molecular markers to distinguish cultivars, subspecies, and wild banana accessions [21,32,35,36,37]. In the presence of noncoding DNA, some of the detected AFLP loci may simply show the signature of selection because they only are associated with the target [43]. The genome scan of banana cultivars from Jazan in Saudi Arabia offers an opportunity to uncover molecular markers for the selected cultivars even though the location and function of the detected outlier loci are uncertain. A reduced representation library of these cultivars’ genomes can be constructed using the AFLP primers used to amplify the outlier loci [44]. This perspective can help to thoroughly study those loci in nature and identify their role in the domestication of banana plants and cultivars.

## Figures and Tables

**Table 1:** Banana cultivars ID, names, species, and sampling locations from Jazan province.

Sample ID	Cultivar Name	Species Name	Location
Ban01	Red Banana	*Musa acuminata*	Fifa
Ban02	Indian Banana	*Musa acuminata*	Fifa
Ban03	Baladi Banana	*Musa acuminata*	Fifa
Ban04	American Banana	*Musa paradisiaca*	Dhamadh
Ban05	Red Banana	*Musa acuminata*	Dhamadh
Ban06	French Banana	*Musa acuminata*	Dhamadh
Ban07	American Banana	*Musa paradisiaca*	Beesh
Ban08	Indian Banana	*Musa acuminata*	Beesh

**Table 2:** Sequences of primers and adaptors that were used to establish the AFLP-PCR technique.

Type	*EcoRI*	5′-Sequence-3′	*MseI*	5′-Sequence-′3
Adaptors	A1	CTCGTAGACTGCGTACC	A1	GACGATGAGTCCTGAG
	A2	AATTGGTACGCAGTC	A2	TACTCAGGACTCAT
1st PCR	+A	GACTGCGTACCAATTCA	+C	GATGAGTCCTGAGTAC
Selective PCR	+ACA	FAM-GACTGCGTACCAATTCAA	+CTC	GATGAGTCCTGAGCTC
	+AGG	HEX-GACTGCGTACCAATTCAG	+CTA	GATGAGTCCTGAGCTA
	+ATA	CY3-GACTGCGTACCAATTCAA	+CTT	GATGAGTCCTGAGCTT

**Table 3:** Genetic diversity and DNA polymorphism based on AFLP bands.

Parameter/Location	Fifa (Ban01–03)	Dhamadh (Ban04–06)	Beesh (Ban07–08)	Overall
Number of polymorphic bands	862	963	571	1008
Mean effective number of alleles (n_e_)	1.470	1.525	1.390	1.461
Standard deviation (n_e_)	0.010	0.010	0.013	0.006
Mean heterozygosity (H_e_)	0.261	0.292	0.195	0.249
Standard deviation (H_e_)	0.006	0.006	0.006	0.003

**Table 4:** Jaccard dissimilarity genetic distance matrix between all the eight Banana samples.

Jaccard *	Ban01	Ban02	Ban03	Ban04	Ban05	Ban06	Ban07	Ban08
Ban01	0	0.571	0.636	0.528	0.492	0.761	0.522	0.541
Ban02	0.571	0	0.62	0.565	0.56	0.755	0.577	0.483
Ban03	0.636	0.62	0	0.642	0.618	0.71	0.647	0.636
Ban04	0.528	0.565	0.642	0	0.517	0.773	0.447	0.551
Ban05	0.492	0.56	0.618	0.517	0	0.75	0.513	0.532
Ban06	0.761	0.755	0.71	0.773	0.75	0	0.812	0.762
Ban07	0.522	0.577	0.647	0.447	0.513	0.812	0	0.556
Ban08	0.541	0.483	0.636	0.551	0.532	0.762	0.556	0

* The red-to-green gradient reflects the minimum to the maximum genetic distance between 0.00 to 1.00, respectively.

**Table 5:** Genetic differentiation through AMOVA of banana samples based on the AFLP loci dataset.

Dataset	Comparison Scheme	Groups	Among Groups (Va)	Within Groups (Vb)	ΦST (*p* < 0.00)
All AFLP	Among locations	3	7%	93%	0.07
	Among cultivars	4	28.95%	71.05%	0.28
Outliers	Among cultivars	4	100%	0%	1.00

## Data Availability

Not applicable.

## Supplementary Materials

The following supporting information can be downloaded at: https://www.mdpi.com/article/10.3390/cimb45030116/s1, Table S1: Band scored by AFLP in eight banana cultivars from Jazan province; Table S2: Loci under selection analysis of the filtered AFLP dataset among the eight Banana samples from Jazan, Saudi Arabia.
